# Supplementary figures and images for: Involvement of the arachidonic acid cytochrome P450 epoxygenase pathway in the proliferation and invasion of human multiple myeloma cells
Source: PeerJ. 2016 Apr 11;4:e1925. doi: 10.7717/peerj.1925 (PMC4830247; doi:10.7717/peerj.1925)

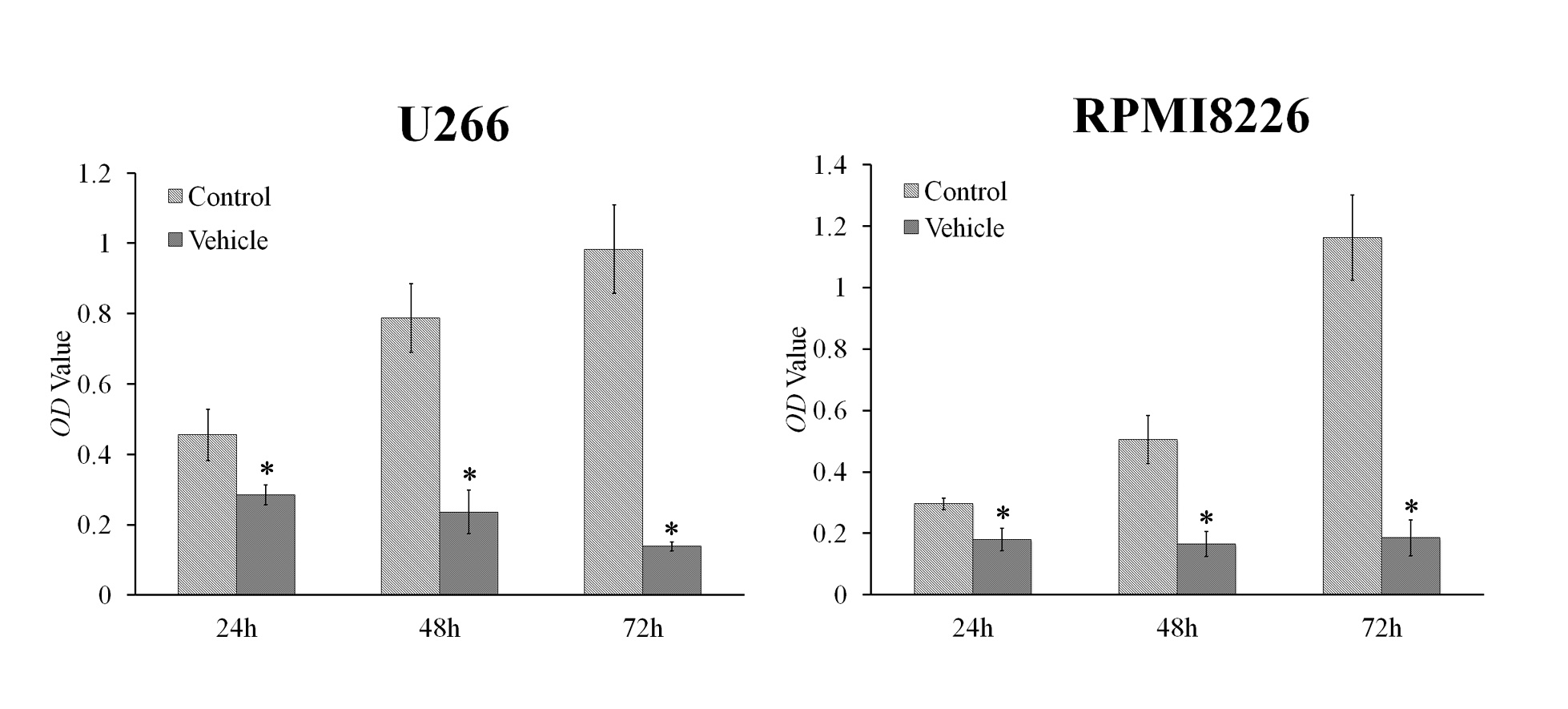

Supplement: Figure S1 — DMSO showed significant inhibitory effect on the viability of U266 (A) and RPMI 8226 (B) cells when used as a vehicle for EETs. [file peerj-04-1925-s001.jpg]
